# Polyphenolic Composition and Antioxidant Activity of *Uncaria tomentosa* Commercial Bark Products

**DOI:** 10.3390/antiox8090339

**Published:** 2019-08-23

**Authors:** Mirtha Navarro, Elizabeth Arnaez, Ileana Moreira, Alonso Hurtado, Daniela Monge, Maria Monagas

**Affiliations:** 1 Department of Chemistry, University of Costa Rica (UCR), Sede Rodrigo Facio, San Pedro de Montes de Oca, San José 2060, Costa Rica; 2 Department of Biology, Technological University of Costa Rica (TEC), Cartago 7050, Costa Rica; 3 Department of Civil Engineering, University of Toronto, 35 St. George Street, Toronto, ON, M5S 1A4, Canada; 4 Department of Economics, State University of New York, SUNY at Binghamton, 400 Vestal Pkwy E, Binghamton, NY 13902, USA; 5 Institute of Food Science Research (CIAL), Spanish National Research Council (CSIC), C/Nicolás Cabrera 9, 28049 Madrid, Spain

**Keywords:** *Uncaria tomentosa*, polyphenols, medicinal herbs, proanthocyanidins, hydroxybenzoic acids, hydroxycinnamic acids, HPLC, TQ-ESI-MS

## Abstract

*Uncaria tomentosa*, which is widely commercialized as an herbal medicine, constitutes an important source of secondary metabolites with diverse biological activities. For instance, we have previously reported, for the first time, of a polyphenolic profile rich in proanthocyanidins from extracts of *U. tomentosa* plants, as well as their antioxidant capacity, antimicrobial activity on aerial bacteria, and cytotoxicity on cancer cell lines. These promising results prompted this research to evaluate the polyphenolic contents of *U. tomentosa* commercial products. We report a detailed study on the polyphenolic composition of extracts from *U. tomentosa* bark products (*n* = 18) commercialized in Costa Rica and Spain. Using HPLC-DAD/TQ-ESI-MS, a total of 25 polyphenolic compounds were identified, including hydroxybenzoic and hydroxycinnamic acids, flavan-3-ol monomers, procyanidin dimers, procyanidin trimers, as well as propelargonidin dimers. Our findings on the polyphenolic profile for all commercial samples show analogous composition to previous reports on *U. tomentosa* bark material, for instance a 41–49% content of procyanidin dimers and the presence of propelargonidin dimers (8–15%). However, most of the 18 commercial samples exhibit low proanthocyanidin contents (254.8–602.8 µg/g), more similar to previous *U. tomentosa* inner bark reports, while some exhibit better results, with one sample (SP-2) showing the highest contents (2386.5 µg/g) representing twice the average value of all 18 commercial products. This sample also exhibits the highest total phenolics (TP) and total proanthocyanidins (PRO) contents, as well as the highest Oxygen Radical Absorbance Capacity (ORAC) value (1.31 µg TE/g). One-way Analysis of Variance (ANOVA) with a Tukey post hoc test indicated significant difference (*p* < 0.05) between products from Costa Rica and Spain for TP and PRO findings, with samples from Spain exhibiting a higher average value. In addition, Pearson correlation analysis results showed a positive correlation (*p* < 0.05) between TP, PRO, and ORAC results, and an especially important correlation between ORAC antioxidant values and procyanidin dimers (*r* = 0.843, *p* < 0.05), procyanidin trimers (*r* = 0.847, *p* < 0.05), and propelargonidin dimers (r = 0.851, *p* < 0.05) contents. Finally, Principal Component Analysis (PCA) results indicated some variability in the composition regardless of their origin. However, only one sample (SP-2) stands out significatively, showing the highest PC1 because of its particularly high proanthocyanidins contents, which could be attributed to the 15% bark polyphenolic extract labeled in this commercial product, which differentiate this sample from all other 17 commercial samples. Therefore, our findings confirmed previous results on the value of extracts in the elaboration of potential commercial products from *U. tomentosa,* rich in proanthocyanidins and exhibiting high antioxidant activity.

## 1. Introduction

*Uncaria tomentosa* L. is a creeper vine from the Rubiaceae family, growing mainly in the rainy tropical forest from Central and South America, and traditionally used as a medicinal plant [1]. Among the compounds isolated from *U. tomentosa*, commonly known as cat’s claw, earlier ones include oxindole alkaloids and triterpenes [2,3], later quinic acid esters [4], and more recently polyphenols including phenolic acids and proanthocyanidins [1,5,6]. Scientific reports account for a wide range of biological activities, including immunostimulant, anti-inflammatory, antioxidant properties, and protective effects against cancer [2,3,7].

Several studies attributed these properties to the presence of alkaloids [2]. For instance, it was reported that *U. tomentosa* pentacyclic oxindole alkaloids (POA) stimulate endothelial cells in vitro to produce a lymphocyte-proliferation-regulating factor [8], while another study reported a better anti-inflammatory effect in mice when using a *U. tomentosa* extract rich in POA with respect to an extract with low POA contents [9]. Similar positive anti-inflammatory effects were reported in the treatment of rheumatoid arthritis patients with POA rich extracts [10]. On the other hand, some reports indicate bioactivity linked with *U. tomentosa* extracts rich in quinic acid esters [4]. For instance, enhanced DNA repair and immune function were reported in rats [11] while inhibition was found on proliferation of normal mouse T and B lymphocytes, and it was proposed that a retarded cell cycle progression caused such inhibition [12].

Recent studies indicate that phenolic compounds present in *U. tomentosa* are at least partially responsible for some of these health effects and point to synergistic interactions among different metabolites [7]. For instance, antioxidant properties have been attributed to extracts containing both alkaloids and polyphenols [1] and more specifically flavan-3-ol monomers [13], with antioxidant values increasing with higher polyphenolic contents. Another report suggested hydroxycinnamic acids and low molecular weight proanthocyanidins were responsible for potent radical scavenging activity [5], providing evidence for an antioxidant mechanism underlying the anti-inflammatory activity of cat’s claw. High antioxidant activity has also been found for *U. tomentosa* bark and leaves extracts rich in hydroxybenzoic acids and proanthocyanidins, including procyanidins, flavalignans, and propelargonidins [14], with a positive correlation between proanthocyanidin contents and antioxidant capacity.

Polyphenols constitute an important group for further studies since they modulate pathways related to chronic inflammation [15] and pro-inflammatory gene expression, as well as having multiple molecular targets [16]. Proanthocyanidins have the advantage over flavonoids of not acting as pro-oxidants as shown by autooxidation studies and because of their important antioxidant properties are suggested to be cancer protective [17]. For instance, proanthocyanidins from grape extracts exert strong free radical scavenging and chelating activities and inhibit lipid oxidation in various cell models in vitro. These antioxidants are promising against a broad range of cancer cells by targeting epidermal growth factor receptor and its downstream pathways, inhibiting over-expression of COX-2 and prostaglandin E2 receptors, or modifying estrogen receptor pathways, resulting in cell cycle arrest and apoptosis [18]. For instance, *U. tomentosa* extracts have been found to exert antiproliferative activity on MCF7 breast cancer cells [19], as well as on AGS gastric and SW620 colon adenocarcinoma cells, exhibiting a positive correlation with procyanidin and propelargonidin contents regarding both antitumoral effect and selectivity in respect of normal cells [20].

On the other hand, phenolic acids are recognized as antimicrobial agents on certain human bacteria, for instance, hydroxybenzoic and hydroxycinnamic acids have shown antimicrobial effects against *Salmonella* [21] while monomeric flavanols inhibit the growth of pathogen bacteria such as *Clostridium dificile* and *Bacteroides* spp. [22]. On their turn, proanthocyanidins are found to produce a change in microbial population towards Gram-negative species (i.e., Enterobacteriaceae), while higher molecular weight polyphenols have greater susceptibility to inhibit Gram-positive bacteria [23]. Specific studies on *U. tomentosa* bark extracts rich in proanthocyanidins, including dimers and oligomers up to undecamers, indicated an antimicrobial effect against respiratory pathogens *Enterococcus faecalis*, *Pseudomonas aeruginosa*, and *Staphylococcus aureus* [14].

As described, these polyphenols, including proanthocyanidins, constituted by condensed flavan-3-ols units, have been the object of different studies due to their potential health benefits, mainly considering their antioxidant activity and the important role they may play by acting as cancer chemo preventive agents. However, their characterization as well as the determination of the content and distribution in botanicals, herbal medicines, and dietary supplements remains a very difficult task [24]. Further, although *U. tomentosa* is widely commercialized as an herbal medicine, commercial products have been scarcely studied and there are no published reports on polyphenolic determinations using modern analytical techniques such as mass spectrometry.

Therefore, considering all these facts, the objective of the present work was to obtain extracts of *U. tomentosa* as commercially available products in Costa Rica and Spain (*n* = 18), in order to determine their detailed polyphenolic contents using HPLC-DAD/TQ-ESI-MS for the characterization of 25 polyphenols, comprising seven hydroxybenzoic acids, three hydroxycinnamic acids, and fifteen proanthocyanidins, including (+)-catechin and (−)-epicatechin flavan-3-ol monomers, seven procyanidin type-B dimers, two procyanidin type-B trimers, as well as four propelargonidin dimers. The quantification of these polyphenols was to be determined in order to compare the commercial products’ composition. Finally, the determination of the products’ antioxidant capacity by Oxygen Radical Absorbance Capacity (ORAC) was to be assessed to explore possible relationships with their proanthocyanidin contents.

## 2. Materials and Methods

### 2.1. Commercial Products, Chemicals, and Reagents

A total of 32 *U. tomentosa* products were purchased in the Spanish Herbalists in Madrid and in the Costa Rican Macrobioticas in San Jose. The products were classified upon their labeled material (bark or leaves) and presentation (capsules, tablets, syrups, or tea bags). In order to be able to obtain valid comparable results, the total of 18 bark capsule products were selected, corresponding to 6 from Costa Rica (CR-1 to CR-6) and 12 from Spain (SP-1 to SP-12). Noteworthy, most products did not specify their composition, one exception was SP-2 whose label specified 15% of polyphenolic bark extract per capsule. The material studied was preserved in a fresh place at room temperature. Solvents such as ethanol, methanol, and acetonitrile were purchased from Baker (Center Valley, PA, USA). Reagents such as AAPH (2,2-azobis(2-amidinopropane) dihydrochloride), sodium molibdate, gallic acid, Trolox (6-hydroxy-2,5,7,8-tetramethylchroman-2-carboxylic acid), fluorescein, and sodium tungstate were provided by Sigma-Aldrich (St. Louis, MO, USA).

### 2.2. Extraction of Phenolic Compounds from Commercial Products of U. tomentosa

To determine the best conditions for extraction of *U. tomentosa* commercial samples, direct extraction with MeOH:H_2_O (80:20, *v*/*v*) was performed for SP-2 as well as sequential extraction with hexane followed by MeOH:H2O (80:20, *v*/*v*). Both extraction processes were carried out in an Accelerated Solvent Extraction (ASE), Dionex™ASE™300 Accelerated Solvent Extractor (Thermo Scientific™, Walthman, MA, USA). ASE parameters for 0.5 g of the sample were the following: 1500 psi, 80 °C, and 3 cycles of 5 min pre-heating time, 5 min heating time, and 8 min static time. Extraction efficiency was determined through total polyphenolics evaluation (TP) and total flavan-3-ols evaluation (PRO), as described in Section 2.3 and Section 2.4. These same ASE conditions were applied for the Factorial Design (FD) performed to further optimize the extractions. Three solvent proportions (70, 80, and 90% MeOH in water, *v*/*v*) and three conditions of temperature at 60, 80 and 100 °C were used in one block, seven experiments, including three central per block. The sequence of the experiments is summarized in Section 3.1. The efficiency of the extractions was determined through three responses, namely total polyphenolics assessment (TP), total flavan-3-ols (PRO), and antioxidant capacity (ORAC) evaluation, as described in Section 2.3, Section 2.4, and Section 2.5. Once the optimal conditions were established, *U. tomentosa* commercial samples (*n* = 18) were extracted in a Dionex™ASE™300 Accelerated Solvent Extractor (Thermo Scientific™, Walthman, MA, USA) using MeOH:H_2_O (80:20, *v*/*v*) at 80 °C, under ASE parameters as described above. Afterwards, the extracts were evaporated to dryness using a Buchi™215 (Flawil, Switzerland) rotavapor.

### 2.3. Determination of Total Phenolic Content

Total polyphenolic contents were evaluated through a variation of the Singleton and Rossi method, using the Folin–Ciocalteu (FC) reagent, which consists of a mixture of phosphomolybdic and phosphotungstic acids. As previously described [6,25], the method consists of mixing 0.5 mL of FC reagent and 10 mL of Na_2_CO_3_ (7.5%) with 0.5 mL of *U. tomentosa* extract dissolved in acidified MeOH (0.1% HCl) to assure extract dissolution regardless of the solvent used for plant material extraction. Finally, water was added to complete 25 mL. A blank was prepared similarly but using 0.5 mL of MeOH (0.1% HCl) instead of the extract. Both blank and extract mixtures were left standing in the dark for 1 h, and subsequently absorbance was measured at 750 nm. Absorbance values were extrapolated in a gallic acid calibration curve in order to obtain total polyphenolic (TP) results, which are expressed as mg gallic acid equivalents (GAE)/g of extract. Analyses were performed in triplicate.

### 2.4. Total Proanthocyanidin Determination

Total proanthocyanidin contents was determined by a modification of the Bate–Smith method, which is based on C–C interflavanic bond oxidative cleavage, as described early [14]. Briefly, 0.2 mL of each *U. tomentosa* extract, 20 mL of butanol/HCl (50:50), and 0.54 mM FeSO_4_ were incubated at 90 °C for 1 h. After cooling to 25 °C, butanol-HCl mixture was added to complete 25 mL and the absorbance of each *U. tomentosa* extract mixture was measured at 550 nm against a blank prepared in the same way described for the extract but without heating. Absorbance values were extrapolated in a cyanidin chloride calibration curve in order to obtain total proanthocyanidin (PRO) results, which are expressed as mg of cyanidin chloride equivalents (CE)/g of extract.

### 2.5. Analysis of Phenolic Compounds by UPLC-DAD-ESI-TQ MS

An Acquity PDA eλ photodiode array detector (DAD) coupled with an UPLC system and an Acquity TQD tandem quadrupole mass spectrometer equipped with Z-spray electrospray interface (Waters, Milford, MA, USA) was used as the UPLC-DAD-ESI-TQ MS system in order to analyze the phenolic composition of extracts from *U. tomentosa* commercial products. Separation for each extract was performed on a Waters® BEH C18 column (2.1 × 100 mm; 1.7 μm) at 40 °C and using a flow rate of 0.5 mL/min. Acetonitrile and water acidified with 2% acetic acid were used as solvents. The gradient consisted of solvent A, water:acetic acid (98:2, *v*/*v*), and solvent B, acetonitrile:acetic acid (98:2, *v*/*v*), and was applied as follows [6,26]: 0–1.5 min: 0.1% B, 1.5–11.17 min: 0.1–16.3% B, 11.17–11.5 min: 16.3–18.4% B, 11.5–14 min: 18.4% B, 14–14.1 min: 18.4–99.9% B, 14.1–15.5 min: 99.9% B, 15.5–15.6 min: 0.1% B, and 15.6–18 min: 0.1% B. The DAD was operating at 250–420 nm, at a 20 point/s rate and 1.2 nm resolution. The ESI parameters consisted of a capillary voltage of 3 kV; source temperature of 130 °C; desolvation temperature of 400 °C; cone gas (N_2_) flow rate of 60 L/h; and desolvation gas (N_2_) flow rate of 750 L/h. Finally, the ESI was operated in negative mode. For quantification, data were collected in the multiple reaction monitoring mode (MRM), tracking specific transitions of parent and product ions for each compound, and with the use of external calibration curves. For instance, main transitions correspond to flavan-3-ol monomers, (+)-catechin and (−)-epicatechin), at *m/z* 289/245, to procyanidin dimers at *m/z* 577/289, to propelargonidin dimers at *m/z* 561/289, and finally to procyanidin trimers at *m/z* 865/577. Commercial standards used for optimizations, mass detector parameters and calibration curves, were (+)-catechin, (-)-epicatechin monomers, procyanidin B1 [(−)-epicatechin-(4β → 8)-(+)-catechin], and procyanidin B2 [(-)-epicatechin-(4β → 8)-(-)-epicatechin] dimers, as well as procyanidin C1 [(-)-epicatechin-(4β → 8)-(-)-epicatechin-(4β → 8)-(-)-epicatechin] trimer. Assignment of other procyanidin structures such as B3 [(+)-catechin-(4α → 8)-(+)-catechin] dimer and B5 [(−)-epicatechin-(4β → 6)-(−)-epicatechin] dimer, was carried out with standards isolated from other plants and using MS/MS spectrum confirmation. In the case of propelargonidin dimers, due to the absence of commercial standards, MS/MS spectrum was performed to the molecular ion at *m*/*z* 561 to confirm their structure. Quantification for these molecules was carried out using the calibration curve of (−)-epicatechin and quantification of unknown procyanidin dimers was performed using the B1 dimer calibration curve. The limit of detection (LOD) and the limit of quantification (LOQ) of the above-mentioned standards were previously published [27,28]. The analyses were performed in triplicate.

### 2.6. Oxygen Radical Absorbance Capacity (ORAC)

The radical scavenging activity of the *U. tomentosa* extracts was determined by the ORAC method as previously described [14,29] using fluoresceine as the fluorescence probe. A Polarstar Galaxy plate reader (BMG Labtechnologies GmbH, Offenburg, Germany) with 485-P excitation and 520-P emission filters was used. Black 96-well untreated microplates (Nunc, Denmark) were used for fluorescence measurements in the Polarstar equipment controlled by Fluostar Galaxy software (v.4.11-0, BMG Labtechnologies GmbH, Offenburg, Germany). The reaction mixture placed in each well consisted of 200 µL of a mixture of fluorescein (70 nM, used as a fluorescence probe), AAPH (12 mM), and antioxidant: Either Trolox (1–8 µM) or *U. tomentosa* extract (at different concentrations). APPH and Trolox solutions were freshly prepared. Fluorescein was freshly diluted from a stock solution (1.17 mM) using 75 mM phosphate buffer (pH 7.4). The reaction was performed at 37 °C. The plate was automatically shaken before the first reading and afterwards fluorescence was measured every minute for 98 min. The curve of the blank (with no antioxidant) was used to normalize the fluorescence measurements and, from the fluorescence normalized curves, the area under the decay curve (AUC) was calculated as follows:

$$AUC=1+\;\overset{i\;=\;98}{\underset{i\;=\;1}{\sum }}\int_{i}/\int_{0}$$

where *f*_0_ is the initial fluorescence reading at 0 min and *f*i is the fluorescence reading at time *i*. Finally, the net AUC that corresponds to a sample was calculated using the following formula:

Net AUC = AUC_antioxidant_ − AUC_blank_.

The regression equation between the net AUC and the antioxidant concentration was therefore calculated. The ORAC value was in turn calculated by dividing the slope of the latter equation by the slope of the Trolox line attained for the same assay. Final ORAC values were expressed as mmol of Trolox equivalents (TE)/g of extract. Each reaction mixture was carried out in duplicate, and three independent runs were performed for each sample.

### 2.7. Statistical Analysis

In order to evaluate if the total phenolic contents (TP, PRO) and the phenolic totals and subclasses contents measured by UPLC-DAD/TQ-ESI-MS contribute to the antioxidant activity, a Pearson correlation analysis was carried out between such variables and ORAC values. One-way analysis of variance (ANOVA) with a Tukey post hoc as statistical tests were applied to TP, PRO, and ORAC evaluations to determine significant difference (*p* < 0.05) between samples from Costa Rica and Spain. Finally, Principal Component Analysis (PCA) was applied to summarize the data from *U. tomentosa* commercial extracts (*n* = 18) taking in consideration 28 variables, including 25 individual phenolic contents, TP, PRO, and ORAC. R (version 1.2.1335) was used as the statistical program.

## 3. Results and Discussion

### 3.1. Extraction from Commercial Products of U. tomentosa

The results obtained with the Accelerated Solvent Extraction (ASE) process described in the materials and methods section allowed to determine extraction results of *U. tomentosa* bark commercial material SP-2 with or without defatting with hexane. The efficiency of the extraction was evaluated by measuring the total phenolic contents (TP) and the total procyanidin contents (PRO), as was also described in the materials and methods section. The results summarized in Table 1 show that the defatting process negatively affect the values of TP and PRO content. In fact, a significant difference was found (*p* < 0.05) with lower values obtained when using hexane.

Therefore, an Accelerated Solvent Extraction (ASE) factorial design was performed considering previous results for polyphenols extraction from food products [30]. Factors considered include three different temperature conditions (60, 80, and 100 °C) and three different aqueous methanolic mixtures as solvent (10, 20 and 30% water in methanol, *v*/*v*). The efficiency of the extraction was evaluated by measuring the total phenolic content (TP), the total procyanidin contents (PRO), and the antioxidant activity using the Oxygen Radical Antioxidant Capacity (ORAC) method. The results are summarized in Table 2.

Statistical analysis of these results revealed a non-significant effect (*p* > 0.05) for the solvent (water–methanol percentage) and the temperature, and neither from the interaction between both factors, for all TP, PRO, and ORAC determinations under all different conditions. Considering these results, intermediate conditions of 80% MeOH and 80 °C of temperature were chosen for polyphenolic extractions of *U. tomentosa* commercial products (*n* = 18). The results obtained agree with previous polyphenolic extraction findings for food products rich in proanthocyanidins, which describe a higher yield of extraction using a similar organic–aqueous solvent percentage and an analogous range of temperatures [30].

### 3.2. Total Phenolic Content in Commercial U. tomentosa Extracts

Among 32 *U. tomentosa* commercial products purchased in Costa Rica and Spain, corresponding to different labeled material (bark or leaves) and presentation (capsules, tablets, syrups, tea bags), 18 bark capsule products were selected for analyses in order to obtain valid comparable results. Table 3. summarizes the results for Total Phenolics (TP) content of ASE extracts from *U. tomentosa* bark products (*n* = 18), applying the Folin–Ciocalteau method, as described in Section 2.3.

Results for total phenolic content (TP) show variability, with values as low as 2.86 mg of gallic acid equivalents (GAE)/g extract up to 31.70 mg GAE/g extract. One-way ANOVA followed by Tukey post hoc test indicated significant difference (*p* < 0.05) between samples from Costa Rica and Spain. Products from Spain show the higher values with an average PT contents of 17.68 mg GAE/g compared to results for samples from Costa Rica, which hold an average of 12.36 mg GAE/g, hence 30% lower. At individual level, CR-2 product shows the lowest value (2.86 mg GAE/g) among all 18 samples while SP-2 shows the highest value (31.70 mg GAE/g), followed by SP-4 (25.01 mg GAE/g).

### 3.3. Total Proanthocyanidins Content in Commercial U. tomentosa Extracts

Results for total flavan-3-ols contents (PRO) of ASE extracts from *U. tomentosa* bark commercial products (*n* = 18) assessed as described in Section 2.4, are summarized in Table 4.

Results from total procyanidin evaluation show variability, with values ranging from 1.45 mg cyanidin chloride equivalent (CE)/g sample up to 52.96 mg CE/g extract. One-way ANOVA followed by a Tukey post-hoc test indicated significant difference (*p* < 0.05) between samples from Costa Rica and Spain. Products from Spain show the higher values with an average PRO contents of 24.42 mg CE/g compared to results for samples from Costa Rica, which hold an average of 13.74 mg CE/g, hence 43.71% lower. Concerning individual samples, CR-2 product holds the lowest value (1.45 mg CE/g) among all samples, while SP-2 shows the highest value (52.96 mg CE/g), followed by SP-4 (40.91 mg CE/g).

In summary, a similar trend is found for both TP and PRO determinations, with products from Spain showing higher contents than products from Costa Rica and, at individual level, sample SP-2 followed by SP-4 show the highest values while sample CR-2 shows the lowest contents of all samples for both total phenolics and total flavan-3-ols.

### 3.4. UPLC-DAD/TQ-ESI-MS Analysis of U. tomentosa Polyphenolic Extracts

**Figure 1:**
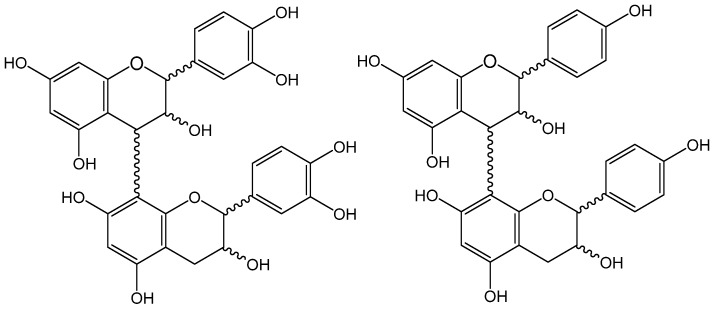
Procyanidin (PC) and propelargonidin (PP) general chemical structures.

The UPLC-DAD/TQ-ESI-MS analysis was performed in the 18 extracts from bark commercial samples of *U. tomentosa*, as described in Section 2.5. The determination of 25 phenolic compounds was achieved, comprising non-flavonoid polyphenols, including seven hydroxybenzoic acids, namely benzoic, 4-hydroxybenzoic, salicylic, gallic, protocatechuic, syringic and vanillic acids; three hydroxycinnamic acids, namely caffeic, *p*-coumaric, and ferulic acids; and, among the flavan-3-ols, both monomers ((+)-catechin and (−)-epicatechin) were found, as well as seven procyanidin dimers, two procyanidin trimers, and four propelargonidin dimers (Figure 1). Results are summarized in Table 5 and Table 6. Multiple reaction monitoring (MRM) transitions under set tandem mass spectrometry (MS/MS) parameters were recorded for compounds found in extracts from *U. tomentosa* bark commercial products. For instance, at *m*/*z* 289/245 for flavan-3-ols monomers ((+)-catechin and (−)-epicatechin), at *m*/*z* 577/289 for procyanidin dimers, at *m*/*z* 561/289 for propelargonidin dimers, and at *m*/*z* 865/577 for procyanidin trimers.

UPLC/MS-MS analysis shows high variability for total phenolic contents with values ranging between 386.9–2924.8 µg/g, and higher contents for *U. tomentosa* commercial samples from Spain (769–2924.8 µg/g) with respect to *U. tomentosa* commercial samples from Costa Rica (386.9–1443.1 µg/g). A similar trend is shown for total contents of flavan-3-ols monomers, procyanidin dimers, procyanidin trimers, and propelargonidin dimers with higher values (56–313.7 µg/g, 358.9–1237 µg/g, 154.6–705.3 µg/g, and 89.3–444.2 µg/g, respectively) for *U. tomentosa* commercial samples from Spain in comparison with samples from Costa Rica (45.2–219.7 µg/g, 160.9–718.4 µg/g, 53.9–296.9 µg/g, and 31.9–178.3 µg/g, respectively), while the opposite trend occurs for the total contents of hydroxycinnamic acids showing higher values for Costa Rica commercial samples (2.4–41.2 µg/g respectively) in comparison with Spain samples (1.9–39.9 µg/g).

The results also show variability in the distribution of polyphenolic compounds. For instance, in the case of hydroxybenzoic acids, protocatechuic acid is the most abundant in all *U. tomentosa* commercial products (28.4–159.4 µg/g), while syringic acid has the lower values (1.6–4.1 µg/g) in all samples. In respect to hydroxycinnamic acids, *p*-coumaric acid is particularly abundant in samples ES-9 and CR-5 with values of 37.2 and 38.4 µg/g, respectively.

Within flavan-3-ols, procyanidin dimers B2 and B4 as well as procyanidin trimer C1 are the ones showing the highest values in all samples (90.1–450.7, 49.3–473.5, and 45.2–526.1 µg/g, respectively), while in the case of propelargonidins, the dimer at 5.65 min is the most abundant (20.8–299.4 µg/g) and the one at 4.43 min shows the less important values (0.8–13.4 µg/g). Finally, among monomers, (−)-epicatechin (42.3–248.2 µg/g) is found in larger concentrations than (+)-catechin (2.9–65.5 µg/g) in all commercial products.

**Figure 2:**
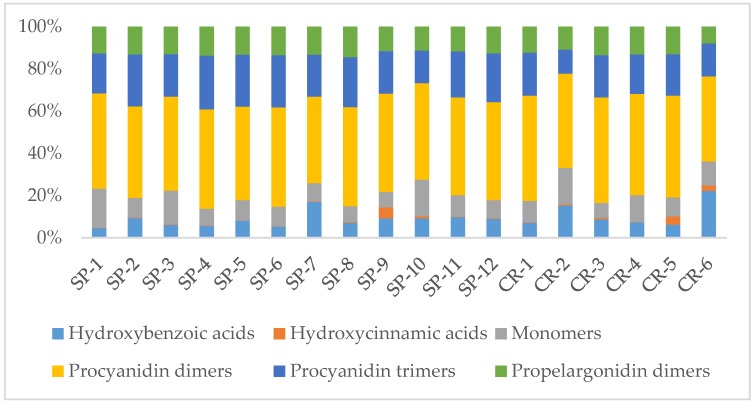
Percentage distribution of polyphenols subclasses by UPLC-DAD/TQ-ESI-MS for *U. tomentosa* commercial bark extracts. CR—samples acquired in Costa Rica, and SP—samples acquired in Spain.

When considering polyphenols subclasses, as illustrated in Figure 2, flavan-3-ols (including monomers, procyanidin dimers, procyanidin trimers, and propelargonidin dimers) are the most abundant group constituting 77.5–94.7% of total contents (300–2652.4 µg/g of extract). Among these flavan-3-ols, procyanidin dimers are the largest group amounting to 41–49% (160.9–1237 µg/g), followed by procyanidin trimers, which constitute 11.4–25.5% (53.9–705 µg/g). Among phenolic acids, hydroxybenzoic acids are the most prominent group with 5.2–18.5% of the total content (70.6–265.5 µg/g), while hydroxycinnamic acids are the least represented with only 0.1–5.2% (1.9–41.2 µg/g).

**Figure 3:**
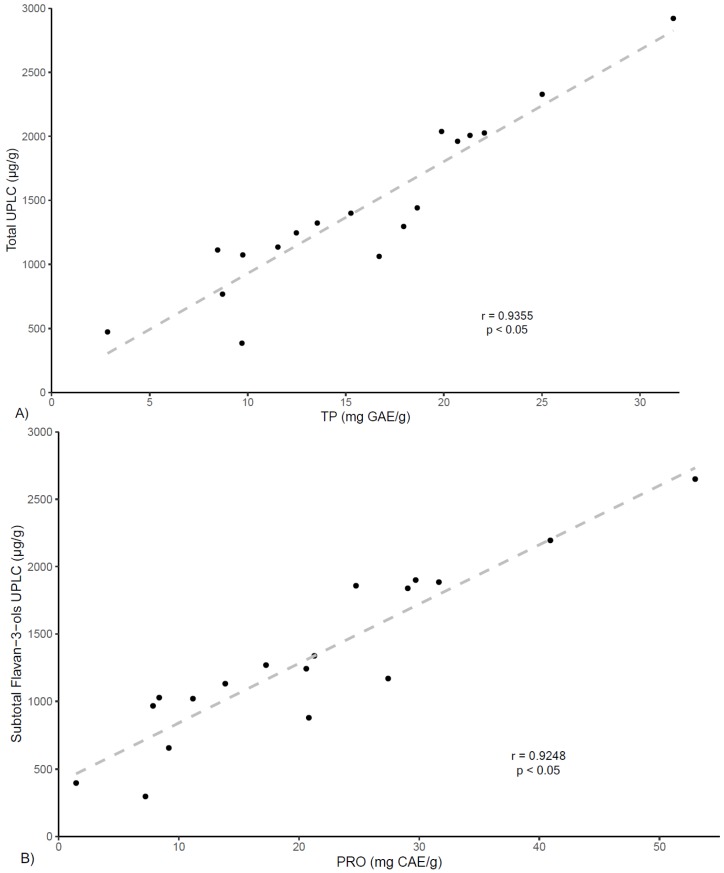
Correlation of (**A**) TP and UPLC total polyphenolic contents and (**B**) PRO and UPLC Flavan-3-ols contents.

When comparing among individual samples, the UPLC analysis revealed, in agreement with TP and PRO determinations, that SP-2 has the highest polyphenolic and flavan-3-ols contents (2924.8 and 2652.4 µg/g extract, respectively), representing twice the average contents of all 18 samples (1445.1 and 1319.3 µg/g respectively). In contrast, the poorest contents for total phenolics and total flavan-3-ols were found for CR-6 (386.9 and 300 µg/g, respectively) and CR-2 (473.2 and 398.9 µg/g, respectively), representing values three times lower than the average for all commercial products, which is also coincident with TP and PRO findings. Finally, as shown in Figure 3, correlation analysis between total polyphenols contents (TP) and the quantification of total phenolics by UPLC demonstrated high correlation (r = 0.936, *p* < 0.05), and likewise, similar results were found for the correlation analysis between PRO values and UPLC flavan-3-ols quantification (r = 0.925, *p* < 0.05).

The above findings on polyphenolic profile for all commercial samples show analogous composition to our previous results on *U. tomentosa* bark material [5], for instance 41–49% contents of procyanidin dimers and the presence of properlagonidin dimers, characteristic of *U. tomentosa* bark extracts. However, most of the 18 commercial samples exhibit proanthocyanidin low contents, similar to *U. tomentosa* inner bark [5], with the exemption of SP-2 sample showing a much higher value (2652.4 µg/g), which could be attributed to the phenolic extract contents labeled in this sample.

### 3.5. Evaluation of Antioxidant Capacity of U. tomentosa Polyphenolic Extracts

In order to perform the evaluation of the antioxidant capacity of *U. tomentosa* bark commercial samples (*n* = 18), the Oxygen Radical Absorbance Capacity (ORAC) methodology was applied, as described in Section 2.5, and results are summarized in Table 7.

The results for the ORAC antioxidant capacity evaluation show the same trend observed for the determination of total polyphenols (TP) and total flavan-3-ols (PRO), with samples commercialized in Spain showing higher values (0.25–1.31 μmol TE/mg of extract) than those from Costa Rica (0.08–0.78 μmol TE/mg). However, one-way ANOVA followed by a Tukey post-hoc test indicated no significant difference (*p* > 0.05) for samples origin, with products from Spain showing a slightly higher average ORAC value of 0.61 μmol TE/mg compared to results for samples from Costa Rica, which hold an average of 0.56 μmol TE/mg. Regarding individual samples, following the same trend than TP and PRO findings, CR-2 has the lowest ORAC value (0.08 μmol TE/mg) among all 18 samples, while SP-2 shows the best value (1.31 μmol TE/mg) followed by SP-4 (1.03 μmol TE/mg).

**Figure 4:**
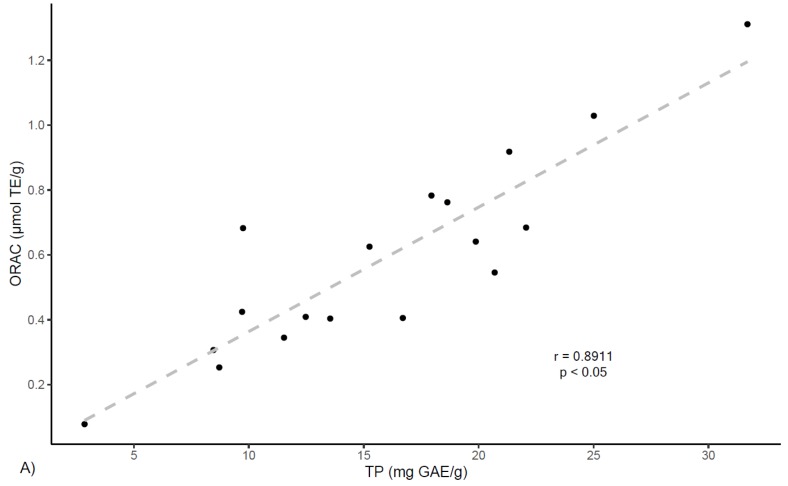

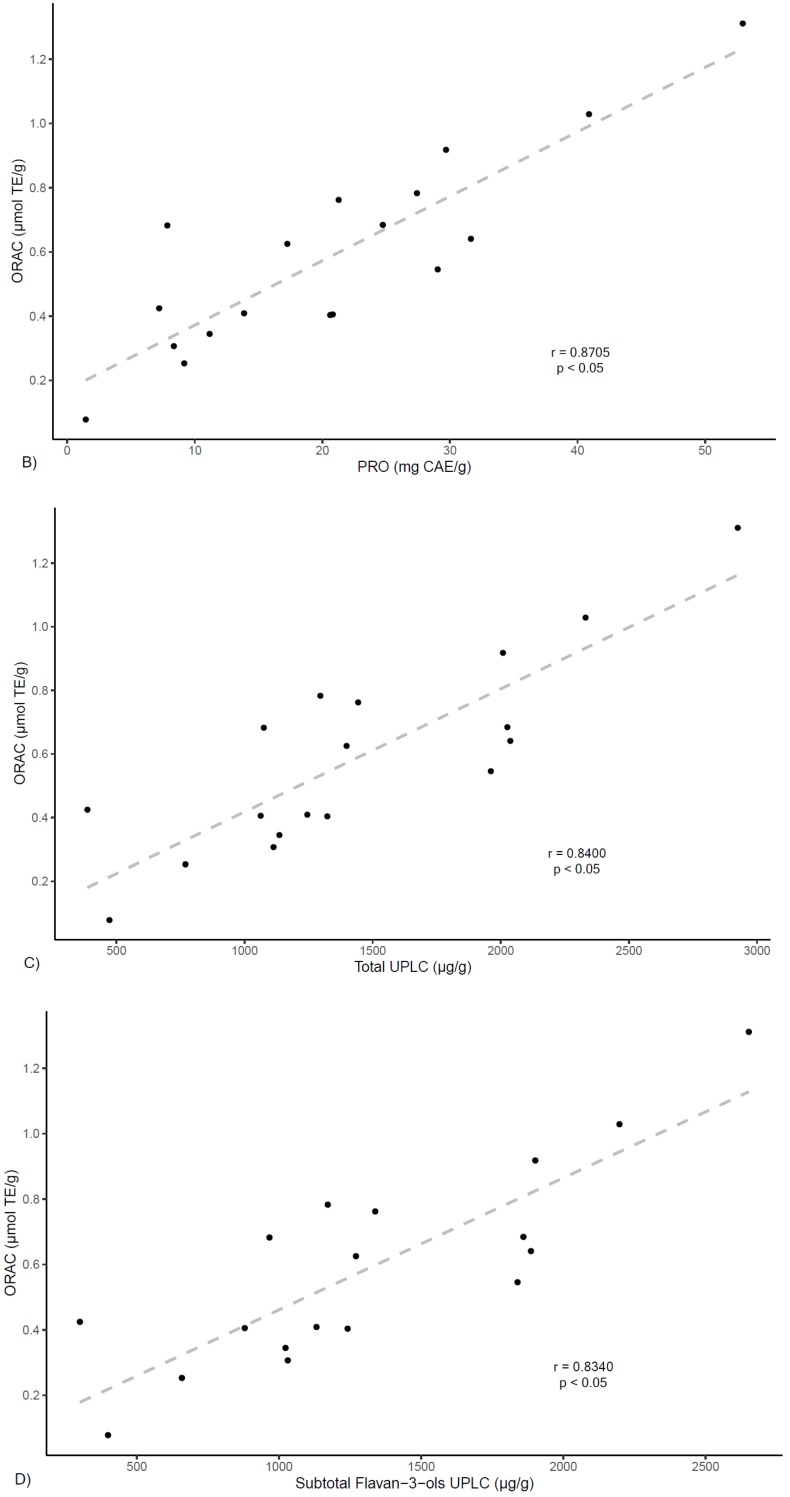
Correlation of antioxidant scavenging activity assessed by the ORAC method with (**A**) Flavan-3-ols by PRO; (**B**) TP by Folin-Ciocalteau; (**C**) Flavan-3-ols by UPLC; and (**D**) TP by UPLC.

On the other hand, correlation analyses were preformed between ORAC antioxidant capacity and extracts phenolic contents. As shown in Figure 4, results show a positive correlation between ORAC values and total phenolics (TP) determined by Folin–Ciocalteau (*r* = 0.891, *p* < 0.05) and by UPLC (*r* = 0.840, *p* < 0.05). In the same manner, ORAC values show positive correlations with total flavan-3-ols PRO findings (*r* = 0.871, *p* < 0.05). These results align with other reports that indicate correlation between total polyphenolic content and ORAC values for several plants [31,32].

**Figure 5:**
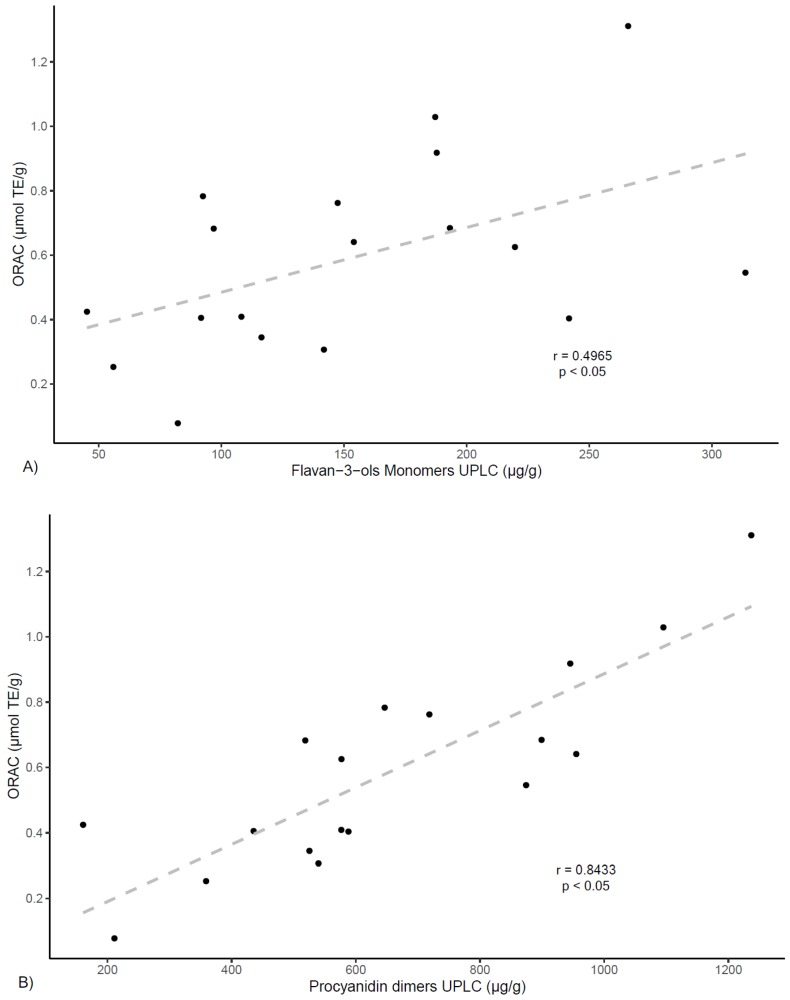

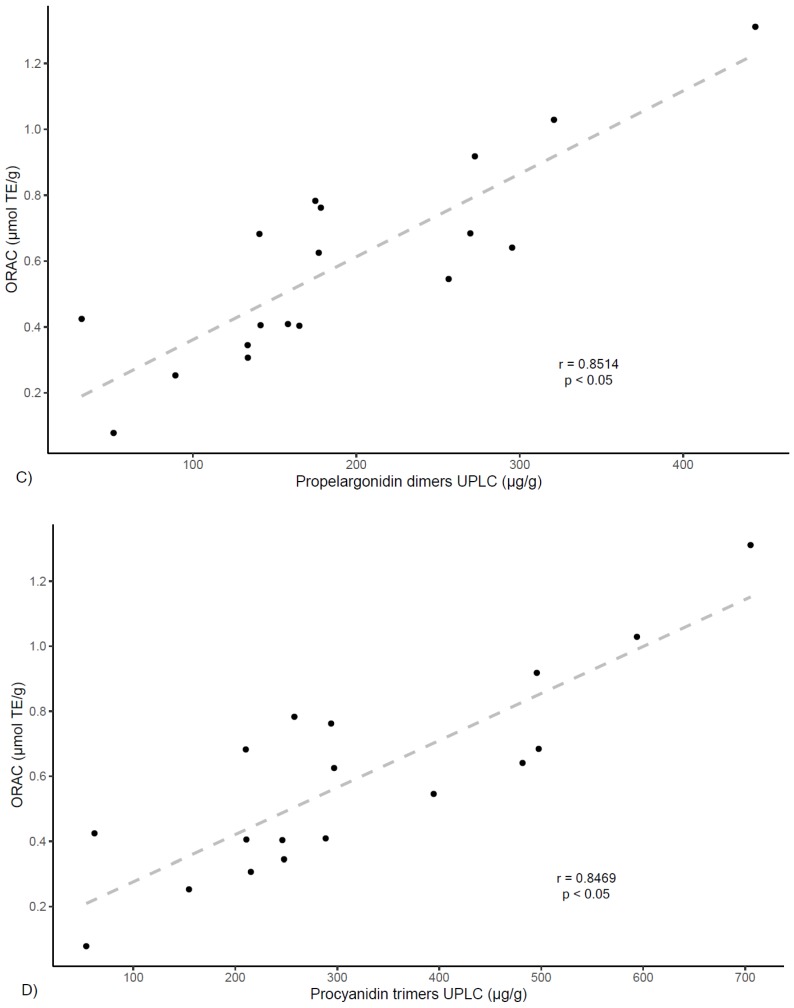
Correlation of antioxidant scavenging activity assessed by the ORAC method with UPLC quantification of flavan-3-ols subgroups: (**A**) monomers; (**B**) procyanidin dimers; (**C**) propelargonidin dimers; and (**D**) procyanidin trimers.

Further, a positive correlation is found between ORAC values and flavan-3-ols determined by UPLC (*r* = 0.834, *p* < 0.05) (Figure 4). For this class of compounds, as shown in Figure 5, findings also show correlation between ORAC values and procyanidin dimers (*r* = 0.843, *p* < 0.05), procyanidin trimers (*r* = 0.847, *p* < 0.05), as well as with propelargonidin dimers (*r* = 0.851, *p* < 0.05), while showing a much lower correlation value for monomers (*r* = 0.496, *p* < 0.05). These results agree with other studies reporting lower antioxidant activity for flavan-3-ol monomers in respect to proanthocyanidins of higher degree of polymerization [33,34] and also align with results from previous studies of polyphenols from *U. tomentosa* plants, suggesting proanthocyanidins may play an important role in higher antioxidant capacity [14,20].

### 3.6. Principal Component Analysis for Polyphenolic Extracts of U. tomentosa Commercial Products

To summarize the results, a statistical Principal Component Analysis (PCA) was performed for *U. tomentosa* commercial bark products (*n* = 18) considering 28 variables, including all 25 individual phenolic compounds, TP, PRO, and ORAC values. Two components (PC1 and PC2) were obtained (loadings > 0.22). The first component (PC1) represented 67.48% of total variance and showed a negative correlation to several proanthocyanidin compounds, including three procyanidin dimers, namely B1, B3, and B dimer at Rt 5.47 mi;, three propelargonidin dimers (at Rt 4.43, 5.65, and 9.27 min); two procyanidin trimers, C1 and B trimer at Rt 4.62 min; as well as TP, PRO, and ORAC values. The second component (PC2) accounted for 9.76% of the total variance and was positively correlated to five phenolic acids, namely 4-hydroxybenzoic acid, p-coumaric acid, ferulic acid, vainillic acid, and syringic acid.

**Figure 6:**
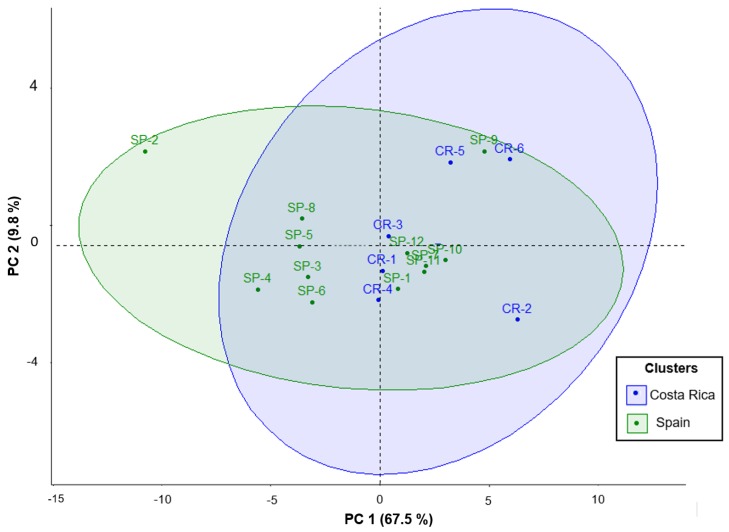
Plane defined by two first principal components (PC1 and PC2) resulting from the PCA analysis of individualized phenolic composition of *U. tomentosa* commercial products (*n* = 18). Origin: CR—Costa Rica, and SP—Spain.

As illustrated in the plane represented by the two components (Figure 6), commercial samples are distributed along PC1, indicating variability for the above-mentioned proanthocyanidins contents. Some samples have particularly low values in PC1, for instance CR-2 and CR-6, which means poorer proanthocyanidins contents and antioxidant values. Meanwhile, SP-2 shows the highest PC1, which agrees with the results discussed in the previous sections, accounting for especially high contents in proanthocyanidins and antioxidant activity among all 18 products. On the other hand, commercial samples are also distributed along PC2, hence showing variability in the hydroxybenzoic and hydroxycinnamic acids contents mentioned above. Particularly, CR-2 shows again the lowest value and SP-2 the highest PC2. Finally, our findings show that although differences were observed in the composition ranges between products from Costa Rica and Spain, only two samples standout significatively, CR-2, which shows the lowest PC1 and PC2, while the opposite is found for SP-2, which shows the highest PC2 and PC1, the latter especially higher because of the rich contents in procyanidins dimers, trimers, and propelargonidin dimers. This last result, as discussed previously, could be attributed to the 15% bark polyphenolic extract labeled in this SP-2 commercial product, which differentiate this sample from all other 17 commercial samples.

In sum, findings for *U. tomentosa* commercial bark products clearly indicate polyphenolic composition analogous to previously reported results for *U. tomentosa* bark material [6,25], however our findings also indicate variability and low proanthocyanidin (procyanidins dimers, trimers, and propelargonidins dimers) contents in most samples, similar to poorer *U. tomentosa* inner bark results [6]. In contrast, a much higher proanthocyanidin content and antioxidant activity was found for the only commercial sample containing polyphenolic extract, which also aligns with our previous results on extracts of *U. tomentosa* material, thus pointing to the advantage of their use for the elaboration of standardized products of *U. tomentosa* as a more valuable source of proanthocyanidins linked to important antioxidant activity and other potential bioactivities [15,20].

## 4. Conclusions

This paper reports valuable information about the phenolic composition of *U. tomentosa* commercial products. Further, this study constitutes the first detailed report of proanthocyanidins in bark capsules (*n* = 18), which are the most widespread *U. tomentosa* commercial product. Using a successful efficient method to obtain enriched polyphenolic extracts from commercial material and advanced analytical techniques such as UPLC/TQ-ESI-MS, a total of 25 phenolic compounds have been identified, including hydroxybenzoic acids, hydroxycinnamic acids, flavan-3-ols monomers, procyanidin dimers, procyanidin trimers, and propelargonidin dimers; these last ones reported for the first time in *U. tomentosa* commercial products. 

Our findings indicate a similar polyphenolic profile for all 18 commercial samples, for instance in procyanidins dimers (41–49%), procyanidin trimers (11.4–25.5%), and especially the presence of propelargonidin dimers (8.2–15.2%), therefore confirming a profile characteristic of *U. tomentosa* authenticated bark material as previously reported [6,25]. However, our findings show much lower contents for total phenolic, total proanthocyanidin, and total phenolic subclasses when compared with former reports for *U. tomentosa* bark [6,25], showing levels analogous to poorer *U. tomentosa* inner bark material [6], therefore suggesting *U. tomentosa* commercial products are a mix of *U. tomentosa* bark and inner bark, instead of solely pulverized bark material.

In addition, results show correlation between ORAC antioxidant activity and proanthocyanidin contents, particularly with procyanidin dimers, procyanidin trimers, and propelargonidin dimers, therefore confirming the potential bioactive value of these extracts due to their proanthocyanidin contents. As discussed, the relationship of these metabolites and their antioxidant capacity with anti-inflammatory effects and cancer chemo-preventive properties, suggest their potential application in the nutraceutical industry [35]. In this context, PCA results showing the best commercial product is the one containing polyphenolic extract, confirm the contribution of proanthocyanidins to *U. tomentosa* product bioactivity.

In sum, this paper corroborates that *U. tomentosa* bark and inner bark material could be a source of proanthocyanidins. However, variability, lower proanthocyanidin contents, and less antioxidant activity would confirm *U. tomentosa* leaves could be a better source [14,20] to be considered for standardized phenolic extracts elaboration. Further studies are needed to determine *U. tomentosa* commercial products potential, for instance purification of extracts and an evaluation of the structure–bioactivity relationship with regard to different bioactivities would be of interest to develop botanical drugs or novel commercial dietary ingredients derived from *U. tomentosa*. For instance, since proanthocyanidins are metabolized by the action of microbiota in the gut [36], it could be particularly valuable to evaluate *U. tomentosa* proanthocyanidins potential health effects in gut-related diseases, such as colon cancer.

## Figures and Tables

**Table 1:** Total phenolic and total flavan-3-ols determination in a commercial sample of *U. tomentosa* for defatting and non-defatting procedures.

Procedure	PT ^1, 3, 4^	PRO ^2, 3, 4^
Hexane and MeOH:H_2_O ^5^	27.93 ^a^ ± 0.25	42.18 ^a^ ± 0.69
MeOH:H_2_O ^6^	30.64 ^b^ ± 0.54	49.82 ^b^ ± 0.54

^1^ mg of gallic acid equivalent/g extract. ^2^ mg cyanidin chloride equivalents/g extract ^3^. Values are expressed as mean ± standard deviation (S.D.). ^4^ Different superscript letters in the same column indicate differences are significant at *p* < 0.05. ^5^ Extraction was performed with hexane and afterwards with 20% aqueous methanol (*v*/*v*). ^6^ Extraction was performed only with 20% aqueous methanol (*v*/*v*).

**Table 2:** Total Phenolics (TP), flavan-3-ols (PRO), and antioxidant activity (ORAC) determination in a commercial sample of *U. tomentosa* under various solvent and temperature conditions.

Experiment	MeOH:H_2_O	T (°C)	TP ^1,4,5^	PRO ^2,4,5^	ORAC ^3,4,5^
1	90:10	100	32.4^a^ ± 1.8	45.2 ^a^ ± 1.9	1.47 ^a^ ± 0.26
2	90:10	60	31.8 ^a^ ± 2.1	48.3 ^a^ ± 1.4	1.14 ^a^ ± 0.24
3	70:30	60	32.1 ^a^ ± 1.8	41.1 ^a^ ± 5.3	1.20 ^a^ ± 0.22
4	70:30	100	32.7 ^a^ ± 3.3	46.7 ^a^ ± 5.5	1.08 ^a^ ± 0.09
5	80:20	80	32.4 ^a^ ± 0.1	47.2 ^a^ ± 2.1	1.47 ^a^ ± 0.27
6	80:20	80	31.7 ^a^ ± 2.8	49.1 ^a^ ± 4.8	1.39 ^a^ ± 0.36
7	80:20	80	31.8 ^a^ ± 2.6	47.2 ^a^ ± 5.2	1.54 ^a^ ± 0.04

^1^ mg of gallic acid equivalent/g; extract ^2^. mg cyanidin chloride equivalents/g; extract ^3^. µmol TE/mg extract ^4^. Values are expressed as mean ± standard deviation (S.D.). ^5^ Different superscript letters in the same column indicate differences are significant at *p* < 0.05.

**Table 3:** Total phenolic (TP)content for extracts of *U. tomentosa* commercial products.

Product	TP (mg/g) ^1,2,3^	Product	TP (mg/g) ^1,2,3^
CR-1	18.64 ^cde^ ± 1.42	SP-4	25.01 ^b^ ± 2.75
CR-2	2.86 ^j^ ± 0.22	SP-5	22.07 ^bc^ ± 1.69
CR-3	17.95 ^cde^ ± 2.23	SP-6	21.33 ^bc^ ± 1.18
CR-4	15.26 ^efg^ ± 0.68	SP-7	16.70 ^def^ ± 0.48
CR-5	9.75 ^hi^ ± 0.38	SP-8	19.88 ^cd^ ± 0.80
CR-6	9.71 ^hi^ ± 0.05	SP-9	8.72 ^i^ ± 0.40
SP-1	13.54 ^fgh^ ± 1.53	SP-10	8.47 ^i^ ± 0.30
SP-2	31.70 ^a^ ± 3.84	SP-11	11.53 ^ghi^ ± 1.29
SP-3	20.70 ^cd^ ± 0.71	SP-12	12.48 ^fghi^ ± 0.29

^1^ mg of gallic acid equivalent/g sample. ^2^ Values are expressed as mean ± standard deviation (S.D.). ^3^ Different superscript letters indicate differences are significant at *p* < 0.05.

**Table 4:** Total flavan-3-ols (PRO) for extracts of *U. tomentosa* commercial products.

Product	PRO (mg/g) ^1,2,3^	Product	PRO (mg/g) ^1,2 3^
CR-1	21.28 ^fg^ ± 0.74	SP-4	40.91 ^b^ ± 0.61
CR-2	1.45 ^l^ ± 0.11	SP-5	24.74 ^ef^ ± 2.71
CR-3	27.42 ^de^ ± 0.48	SP-6	29.70 ^cd^ ± 0.40
CR-4	17.25 ^hi^ ± 0.14	SP-7	20.82 ^fgh^ ± 1.25
CR-5	7.85 ^k^ ± 0.53	SP-8	31.64 ^cd^ ± 0.65
CR-6	7.21 ^k^ ± 0.67	SP-9	9.16 ^k^ ± 0.36
SP-1	20.61 ^gh^ ± 2.05	SP-10	8.36 ^k^ ± 0.59
SP-2	52.96 ^a^ ± 2.67	SP-11	11.17 ^jk^ ± 1.04
SP-3	29.05 ^cd^ ± 3.16	SP-12	13.87 ^ij^ ± 1.31

^1^ mg cyanidin chloride equivalents/g sample. ^2^ Values are expressed as mean ± standard deviation (S.D.). ^3^ Different superscript letters indicate differences are significant at *p* < 0.05.

**Table 5:** Phenolic composition of *U. tomentosa* bark commercial extracts.

Sample	CR-1	CR-2	CR-3	CR-4	CR-5	CR-6	SP-1	SP-2	SP-3
Compound	Concentration (µg/g Extract) ^1^								
*Hydroxybenzoic acids*									
Benzoic acid	4.2 ± 0.1	20.9 ± 1.0	1.4 ± 0.1	13.9 ± 0.8	10.3 ± 1.0	10.1 ± 0.0	14.8 ± 0.3	27.0 ± 0.4	25.1 ± 2.5
Salicylic acid	5.6 ± 0.0	2.2 ± 0.0	4.2 ± 0.0	8.5 ± 0.9	5.2 ± 0.0	5.8 ± 0.2	10.0 ± 0.3	21.8 ± 0.0	9.8 ± 0.3
4-hydroxybenzoic acid	6.9 ± 0.4	3.0 ± 0.1	6.9 ± 0.2	5.6 ± 0.5	9.9 ± 0.3	9.9 ± 0.3	6.2 ± 0.3	13.8 ± 0.3	6.8 ± 0.0
Protocatechuic acid	65.5 ± 0.3	35.3 ± 0.5	84.9 ± 0.6	64.9 ± 5.2	28.4 ± 0.6	30.1 ± 3.9	31.6 ± 2.2	159.4 ± 6.6	54.9 ± 0.2
Gallic acid	9.4 ± 0.2	4.3 ± 0.3	8.8 ± 0.7	21.2 ± 1.4	1.4 ± 0.0	2.1 ± 0.1	8.4 ± 0.2	20.7 ± 0.2	8.4 ± 0.6
Vainillinic acid	7.8 ± 0.3	4.6 ± 0.5	5.6 ± 0.6	8.6 ± 0.4	9.4 ± 0.9	9.8 ± 0.7	5.1 ± 0.3	18.7 ± 0.6	11.2 ± 0.3
Syringic acid	2.6 ± 0.0	1.6 ± 0.2	4.1 ± 0.3	2.7 ± 0.3	2.5 ± 0.2	3.6 ± 0.1	3.4 ± 0.0	4.1 ± 0.2	3.3 ± 0.1
*∑ Hydroxybenzoic acids*	102.0	71.9	115.9	125.4	67.1	71.4	79.5	265.5	119.5
*Hydroxycinnamic acids*									
*p*-cumaric acid	0.6 ± 0.1	0.7 ± 0.0	0.9 ± 0.0	1.2 ± 0.1	38.4 ± 0.1	12.7 ± 1.1	1.3 ± 0.0	1.8 ± 0.0	1.3 ± 0.1
Caffeic acid	1.6 ± 0.0	0.9 ± 0.1	6.5 ± 0.6	0.7 ± 0.0	0.8 ± 0.1	0.7 ± 0.1	0.4 ± 0.0	4.5 ± 0.2	1.1 ± 0.1
Ferulic acid	0.7 ± 0.0	0.8 ± 0.1	0.5 ± 0.0	0.5 ± 0.0	2.0 ± 0.1	2.1 ± 0.2	0.6 ± 0.0	0.6 ± 0.0	0.6 ± 0.0
*∑ Hydroxycinnamic acids*	2.9	2.4	7.9	2.4	41.2	15.5	2.3	6.9	3.0
*Flavan-3-ols: monomers*									
(+)-Catechin	16.1 ± 0.7	7.7 ± 0.6	8.3 ± 0.1	28.7 ± 1.9	7.0 ± 0.1	2.9 ± 0.2	39.8 ± 4.0	42.4 ± 0.2	65.5 ± 0.5
(−)-Epicatechin	131.3 ± 2.7	74.6 ± 1.8	84.2 ± 3.1	191.0 ± 10.2	89.9 ± 2.0	42.3 ± 1.1	202.0 ± 20.2	223.5 ± 1.7	248.2 ± 2.1
*∑ Monomers*	147.4	82.3	92.5	219.7	96.9	45.2	241.8	265.9	313.7
*Flavan-3-ols: procyanidin dimers*									
Procyanidin B1	20.9 ± 0.8	4.6 ± 0.5	17.3 ± 0.8	20.6 ± 2.9	12.9 ± 1.4	3.1 ± 0.0	19.8 ± 2.0	59.2 ± 3.6	38.1 ± 1.8
Procyanidin B2	296.1 ± 8.4	114.3 ± 1.6	264.9 ± 11.0	213.8 ± 13.5	232.9 ± 4.1	90.1 ± 2.2	219.6 ± 22.0	450.7 ± 26.2	377.4 ± 5.7
Procyanidin B3	17.9 ± 0.9	3.6 ± 0.1	15.3 ± 0.1	13.1 ± 2.1	10.0 ± 0.4	2.1 ± 0.1	19.7 ± 2.0	52.6 ± 2.7	35.5 ± 1.8
Procyanidin B4	288.8 ± 3.5	70.2 ± 1.6	272.3 ± 0.8	235.9 ± 13.8	201.5 ± 0.7	49.3 ± 2.9	248.2 ± 24.8	473.5 ± 15.9	310.1 ± 6.1
Procyanidin B5	71.8 ± 2.3	14.0 ± 0.9	58.4 ± 1.7	71.1 ± 6.9	47.4 ± 1.9	14.6 ± 0.9	63.5 ± 6.4	150.4 ± 10.9	85.5 ± 0.3
Procyanidin B (5.47 min)	16.1 ± 0.8	2.9 ± 0.1	13.0 ± 0.8	15.9 ± 0.2	9.8 ± 0.9	0.0 ± 0.0	13.7 ± 1.4	35.6 ± 3.6	20.8 ± 0.0
Procyanidin B (9.27 min)	6.8 ± 0.4	1.6 ± 0.2	5.3 ± 0.1	6.6 ± 0.1	4.3 ± 0.2	1.7 ± 0.0	3.9 ± 0.4	15.0 ± 0.1	6.9 ± 0.0
*∑ Procyanidin dimers*	718.4	211.2	646.5	577.0	518.8	160.9	588.4	1,237.0	874.3
*Flavan-3-ols: propelargonidin dimers*									
Propelargonidin dimer (4.43 min)	5.6 ± 0.2	2.0 ± 0.2	5.5 ± 0.6	5.6 ± 0.7	4.0 ± 0.4	0.8 ± 0.0	5.3 ± 0.5	13.4 ± 0.5	9.4 ± 0.4
Propelargonidin dimer (5.01 min)	36.3 ± 0.9	10.1 ± 0.5	35.2 ± 0.9	36.1 ± 3.1	27.3 ± 1.4	6.1 ± 0.6	31.3 ± 3.1	63.3 ± 2.9	41.1 ± 0.7
Propelargonidin dimer (5.65 min)	114.4 ± 3.5	35.0 ± 1.0	113.7 ± 5.3	114.0 ± 11.1	94.4 ± 1.8	20.8 ± 0.9	110.1 ± 11.0	299.4 ± 10.9	173.7 ± 4.9
Propelargonidin dimer (9.27 min)	22.0 ± 0.3	4.4 ± 0.1	20.5 ± 0.2	21.3 ± 1.1	15.0 ± 0.6	4.2 ± 0.4	18.4 ± 1.8	68.1 ± 3.5	32.3 ± 1.4
*∑ Propelargonidin dimers*	178.3	51.5	174.9	177.0	140.7	31.9	165.1	444.2	256.5
*Flavan-3-ols: procyanidin trimers*									
Procyanidin C1	229.8 ± 0.5	45.2 ± 2.4	207.1 ± 0.9	223.2 ± 4.5	166.1 ± 4.9	49.5 ± 1.7	196.8 ± 19.7	526.1 ± 52.6	286.7 ± 2.3
Trimer B (4.62 min)	64.3 ± 1.8	8.7 ± 0.1	50.9 ± 0.2	63.7 ± 4.6	44.2 ± 1.9	12.5 ± 1.3	49.5 ± 5.0	179.2 ± 17.9	107.8 ± 10.8
*∑ Procyanidin trimers*	294.1	53.9	258.0	296.9	210.3	62.0	246.3	705.3	394.5
Total	1443.1	473.2	1295.7	1398.4	1075.0	386.9	1323.4	2924.8	1961.5

^1^ Values are expressed as mean ± standard deviation (S.D.).

**Table 6:** Phenolic composition of *U. tomentosa* bark commercial extracts.

Sample	SP-4	SP-5	SP-6	SP-7	SP-8	SP-9	SP-10	SP-11	SP-12
Compound	Concentration (µg/g Extract) ^1^								
*Hydroxybenzoic acids*									
Benzoic acid	12.4 ± 1.0	32.8 ± 3.1	10.1 ± 0.2	14.4 ± 0.4	31.8 ± 3.2	10.1 ± 0.0	3.4 ± 0.1	10.1 ± 1.0	24.8 ± 1.8
Salicylic acid	8.9 ± 0.0	9.4 ± 0.1	7.1 ± 0.1	2.9 ± 0.1	12.5 ± 0.4	6.0 ± 0.0	3.1 ± 0.2	6.2 ± 0.0	9.0 ± 0.2
4-hydroxybenzoic acid	6.1 ± 0.2	8.6 ± 0.4	5.2 ± 0.2	8.9 ± 0.1	7.3 ± 0.3	9.3 ± 0.0	5.0 ± 0.0	6.5 ± 0.4	6.9 ± 0.0
Protocatechuic acid	77.6 ± 0.2	81.5 ± 0.5	63.6 ± 0.8	133.1 ± 1.1	68.8 ± 1.9	30.1 ± 0.4	48.1 ± 1.3	62.0 ± 0.0	47.9 ± 1.1
Gallic acid	14.8 ± 0.2	15.6 ± 0.5	9.6 ± 0.0	12.7 ± 0.5	7.0 ± 0.1	2.5 ± 0.1	5.0 ± 0.1	12.1 ± 0.9	8.1 ± 0.8
Vainillinic acid	9.1 ± 0.1	11.6 ± 1.2	7.2 ± 0.7	7.1 ± 0.7	12.2 ± 1.2	9.5 ± 0.3	7.4 ± 0.2	10.7 ± 0.3	11.6 ± 0.2
Syringic acid	2.5 ± 0.2	3.4 ± 0.2	2.6 ± 0.3	2.6 ± 0.3	3.9 ± 0.4	3.1 ± 0.3	2.9 ± 0.3	2.5 ± 0.2	2.6 ± 0.3
*∑ Hydroxybenzoic acids*	131.4	162.9	105.4	181.7	143.5	70.6	74.9	110.1	110.9
*Hydroxycinnamic acids*									
*p*-cumaric acid	0.6 ± 0.0	1.4 ± 0.0	0.6 ± 0.0	0.6 ± 0.0	5.4 ± 0.5	37.2 ± 2.1	4.1 ± 0.2	1.0 ± 0.0	1.2 ± 0.1
Caffeic acid	1.1 ± 0.0	1.5 ± 0.0	1.0 ± 0.0	1.0 ± 0.1	1.5 ± 0.1	0.6 ± 0.0	2.0 ± 0.0	1.6 ± 0.1	1.0 ± 0.0
Ferulic acid	0.5 ± 0.0	0.5 ± 0.1	0.3 ± 0.0	0.3 ± 0.0	0.7 ± 0.1	2.1 ± 0.1	1.6 ± 0.1	0.4 ± 0.0	0.5 ± 0.0
*∑ Hydroxycinnamic acids*	2.2	3.4	1.9	1.9	7.6	39.9	7.7	3.0	2.7
*Flavan-3-ols: monomers*									
(+)-Catechin	20.5 ± 1.6	27.5 ± 0.4	23.1 ± 1.0	10.0 ± 0.2	13.8 ± 0.6	3.9 ± 0.4	20.4 ± 0.5	11.5 ± 0.1	8.6 ± 0.2
(−)-Epicatechin	166.7 ± 8.8	165.7 ± 8.2	164.7 ± 3.6	81.7 ± 5.9	140.2 ± 5.7	52.1 ± 4.0	121.4 ± 1.5	104.9 ± 1.5	99.6 ± 6.5
*∑ Monomers*	187.2	193.2	187.8	91.7	154.0	56.0	141.8	116.4	108.2
*Flavan-3-ols: procyanidin dimers*									
Procyanidin B1	45.7 ± 2.2	31.0 ± 2.6	33.6 ± 1.3	10.0 ± 0.1	28.8 ± 1.3	6.4 ± 0.4	12.6 ± 0.2	11.7 ± 0.7	13.7 ± 0.0
Procyanidin B2	399.2 ± 37.9	355.6 ± 24.2	370.9 ± 8.8	210.7 ± 10.2	389.2 ± 7.5	160.1 ± 6.0	229.2 ± 6.3	228.3 ± 4.7	254.9 ± 4.7
Procyanidin B3	35.4 ± 1.5	24.3 ± 1.1	26.6 ± 0.0	8.0 ± 0.1	22.4 ± 0.9	4.8 ± 0.1	10.5 ± 0.8	10.2 ± 1.0	10.0 ± 0.0
Procyanidin B4	445.4 ± 24.9	364.2 ± 12.3	374.5 ± 19.2	163.7 ± 6.3	377.1 ± 9.0	146.4 ± 1.3	219.3 ± 2.3	211.8 ± 14.3	227.1 ± 0.3
Procyanidin B5	125.6 ± 7.8	91.5 ± 6.8	106.6 ± 1.3	32.8 ± 0.8	105.8 ± 2.8	31.4 ± 0.1	53.5 ± 0.1	48.3 ± 3.1	54.8 ± 0.4
Procyanidin B (5.47 min)	33.1 ± 3.2	21.9 ± 1.6	24.4 ± 1.0	6.9 ± 0.6	23.5 ± 2.4	6.8 ± 0.0	9.5 ± 0.5	9.5 ± 0.5	9.5 ± 0.5
Procyanidin B (9.27 min)	10.9 ± 0.6	10.7 ± 0.7	8.9 ± 0.9	3.3 ± 0.2	10.3 ± 0.2	3.0 ± 0.3	5.5 ± 0.1	5.7 ± 0.1	6.8 ± 0.4
*∑ Procyanidin dimers*	1095.3	899.2	945.5	435.4	955.1	358.9	540.1	525.5	576.8
*Flavan-3-ols: propelargonidin dimers*									
Propelargonidin dimer (4.43 min)	11.9 ± 0.7	8.4 ± 0.3	9.3 ± 0.2	4.1 ± 0.3	8.5 ± 0.9	2.4 ± 0.2	4.1 ± 0.3	3.8 ± 0.3	4.4 ± 0.1
Propelargonidin dimer (5.01 min)	63.4 ± 0.2	53.2 ± 1.3	54.8 ± 1.1	32.7 ± 2.0	54.9 ± 0.6	20.0 ± 0.5	28.1 ± 0.4	28.6 ± 1.7	30.3 ± 0.9
Propelargonidin dimer (5.65 min)	199.0 ± 2.7	171.5 ± 8.3	170.5 ± 0.6	89.4 ± 2.9	190.5 ± 1.0	56.2 ± 3.9	85.9 ± 0.8	85.9 ± 8.0	104.2 ± 2.5
Propelargonidin dimer (9.27 min)	46.6 ± 4.8	36.7 ± 3.5	37.9 ± 1.2	15.2 ± 1.1	41.5 ± 0.3	10.7 ± 0.1	15.5 ± 0.1	15.2 ± 0.4	19.3 ± 0.2
*∑ Propelargonidin dimers*	320.9	269.8	272.5	141.4	295.4	89.3	133.6	133.5	158.2
*Flavan-3-ols: procyanidin trimers*									
Procyanidin C1	452.0 ± 45.2	383.2 ± 38.3	379.5 ± 38.0	169.6 ± 17.0	372.1 ± 6.7	118.6 ± 6.0	190.6 ± 2.6	193.6 ± 10.2	225.8 ± 9.6
Trimer B (4.62 min)	141.9 ± 14.2	114.4 ± 11.4	116.1 ± 9.5	41.4 ± 4.1	109.7 ± 6.1	36.0 ± 3.6	24.7 ± 1.5	54.2 ± 5.4	62.8 ± 6.0
*∑ Procyanidin trimers*	593.9	497.6	495.6	211.0	481.8	154.6	215.3	247.8	288.6
Total	2330.9	2026.1	2008.7	1063.1	2037.4	769.3	1113.4	1136.3	1245.4

^1^ Values are expressed as mean ± standard deviation (S.D.).

**Table 7:** Oxygen Radical Absorbance Capacity (ORAC) antioxidant activity of extracts from *U. tomentosa* bark commercial products.

Product	ORAC (µmol/g) ^1,2,3^	Product	ORAC (µmol/g) ^1,2,3^
CR-1	0.76 ^cde^ ± 0.04	SP-4	1.03 ^b^ ± 0.04
CR-2	0.08 ^j^ ± 0.01	SP-5	0.69 ^de^ ± 0.06
CR-3	0.78 ^cd^ ± 0.02	SP-6	0.92 ^bc^ ± 0.06
CR-4	0.63 ^def^ ± 0.01	SP-7	0.41 ^gh^ ± 0.01
CR-5	0.68 ^de^ ± 0.02	SP-8	0.64 ^def^ ± 0.04
CR-6	0.43 ^fgh^ ± 0.03	SP-9	0.25 ^hi^ ± 0.02
SP-1	0.40 ^gh^ ± 0.04	SP-10	0.31 ^h^ ± 0.02
SP-2	1.31 ^a^ ± 0.08	SP-11	0.35 ^gh^ ± 0.01
SP-3	0.55 ^efg^ ± 0.03	SP-12	0.41 ^gh^ ± 0.03

^1^ µmol of Trolox equivalents (TE)/mg product. ^2^ Values are expressed as mean ± standard deviation (S.D.). ^3^ Different superscript letters indicate differences are significant at *p* < 0.05.
